# Comparison of HTLV-I Proviral Load in Adult T Cell Leukemia/Lymphoma (ATL), HTLV-I-Associated Myelopathy (HAM-TSP) and Healthy Carriers

**Published:** 2013-03

**Authors:** Mohammad Mehdi Akbarin, Hossein Rahimi, Tahereh HassanNia, Ghazaleh Shoja Razavi, Faezeh Sabet, Abbas Shirdel

**Affiliations:** 1 Inflammation and inflammatory research centre, Medical School, Mashhad University of Medical Science, Mashhad- Iran; 2 Internal medicine Dept. Medical School, Mashhad University of Medical Science, Mashhad- Iran; 3 Internal Medicine Dept, Amir al Moemenin Hospital, Arak University of Medical Sciences.; 4 Navid Medical Lab, Mashhad- Iran

**Keywords:** HTLV-I, HAM/TSP, ATL, HTLV-I proviral load

## Abstract

***Objective(s):*** Human T Lymphocyte Virus Type one (HTLV-I) is a retrovirus that infects about 10-20 million people worldwide. Khorasan province in Iran is an endemic area. The majority of HTLV-I-infected individuals sustain healthy carriers but small proportion of infected population developed two progressive diseases: HAM/TSP and ATL. The proviral load could be a virological marker for disease monitoring, therefore in the present study HTLV-I proviral load has been evaluated in ATL and compared to HAM/TSP and healthy carriers.

***Materials and Methods:*** In this case series study, 47 HTLV-I infected individuals including 13 ATL, 23 HAM/TSP and 11 asymptomatic subjects were studied. Peripheral blood mononuclear cells (PBMCs) were investigated for presence of HTLV-I DNA provirus by PCR using LTR and Tax fragments. Then in infected subjects, HTLV-I proviral load was measured using real time PCR TaqMan method.

***Results:*** The average age of patients in ATL was 52±8, in HAM/TSP 45.52±15.17 and in carrier’s 38.65±14.9 years which differences were not statistically significant. The analysis of data showed a significant difference in mean WBC among study groups (ATL *vs *HAM/TSP and carriers *P=*0.0001). Moreover, mean HTLV-I proviral load was 11967.2 ± 5078, 409 ± 71.3 and 373.6 ± 143.3 in ATL, HAM/TSP and Healthy Carriers, respectively. The highest HTLV-I proviral load was measured in ATL group that had a significant correlation with WBC count (R=0.495, *P=*0.001). The proviral load variations between study groups was strongly significant (ATL vs carrier *P=*0.0001; ATL vs HAM/TSP *P=* 0.0001 and HAM/TSP *vs* carriers *P*< 0.05).

***Conclusion*** ***:*** The present study demonstrated that HTLV-I proviral load was higher in ATL group in comparison with HAM/TSP and healthy carriers. Therefore, HTLV-I proviral load is a prognostic factor for development of HTLV-I associated diseases and can be used as a monitoring marker for the efficiency of therapeutic regime.

## Introduction

Human T Lymphocyte Virus Type one is a RNA virus belongs to retroviruses family (1).It has been estimated that HTLV-I infects 10-20 million people worldwide and in some area is endemic such as the Caribbean basin, South America, Central Africa, southwestern Japan , the Melanesian Islands and the Middle East (2-4). The HTLV-I infection in Khorasan particularly Mashhad, Neyshabour and Sabzevar is endemic and frequency is estimated to be 2-4% of the entire population (3, 5-7). The majority of HTLV-I-infected individuals sustain healthy carriers (8) Whereas small proportion of infected subjects develops the neoplastic and malignancy disorders like adult T-cell leukemia (ATL) and the inflammatory disease such as HTLV-I- Associated Myelopathy/Tropical Spastic Paraparesis (HAM/TSP) and uveitis (9). Adult T cell leukemia/ lymphoma is an aggressive T-cell proliferation of HTLV-I infected cells with a very poor prognosis. The symptoms of ATL are organomegaly, cutaneous lesions, hypercalcemia and leukemia with atypical polylobed lymphocytes(flower liked ) displaying a CD2+, CD3+, CD4+, CD8-, CD7- T-cell phenotype(10, 11). Although 10-20 million are infected by HTLV-I in the world, only 1-4% develops ATL during 7^th^ decade of lifespan. The latency period is more than 30 years (12). Another HTLV-I associated disease is HAM/TSP that is a chronic and insidious neurologic disorders which sometimes presents with impairment of lower limb strength and mild changes in sensation autonomic abnormalities including neurogenic bladder and bowel (13). Many studies demonstrated that interaction between HTLV-I proteins such as Tax and HBZ with host cells biological activities might be implicated in HTLV-I associated manifestations (15) ( Ahmadi*, et al,* 2013 in press). Since HTLV-I integrated into host genome and has a long period of latency, the percentage of infected cells and proviral load might be good prognostic and monitoring markers for development and therapy efficiency of HTLV-I associated diseases. Consequently, the proviral load seems to be valuable virological marker for disease monitoring, however the impact mechanisms of these factors on disease progression have not been established yet (14). In this study, we investigated HTLV-I proviral load in three groups of infected HTLV-I to determine diffractions between two patients’ pollutions and healthy carrier and evaluated the value on HTLV-I proviral load in prognosis of the diseases.

## Materials and Methods

*Laboratory testes*

 Flow cytometry was used for phenotyping T cell markers ( CD2,3,4,7,8) in ATL group that had symptoms such as organomegaly, cutaneous lesions, hypercalcemia and leukemia with atypical polylobed lymphocytes(flower liked ) .Liver function tests including SGOT,SGPT.ALP and LDH were performed and calcium checked routinely for ATL patients.

In HAM/TSP groups Myelopathy proved by electromyography (EMG) and brain radiography. Seropositive activity checked by ELISA commercial kit (Delawer, USA). PBMCs were isolated from whole blood by Ficoll density gradient (Cederline, Ontario, Canada). Then cellular DNA was tested for the presence of HTLV-I provirus by conventional PCR using specific primers for Tax and LTR (6).

*Study population*

A case series study carried out from July 2011 to December 2013 in Mashhad University of Medical Sciences (MUMS) hospitals on 47 HTLV-I infected individuals including 23 HAM/TSP patients; 9 males and 14 females referred by two subspecialists. Thirteen suspected ATL patients investigated, which the disease confirmed in nine individuals according to symptoms and laboratory tests (three males and five females). Six males and five females asymptomatic HTLV-I infected people age matched subjects were selected as healthy carrier. All subjects were seropositive for HTLV-I that then proved by conventional PCR.

*Proviral load measurement*

To assess the HTLV-I proviral load, PBMCs were isolated from EDTA-treated blood samples as explained earlier. Real time PCR was performed using a commercial absolute quantification kit (Novin Gene, Iran) to measure the proviral load of HTLV-I using specific primers and a fluorogenic probe by a Rotorgen Q Real-Time PCR machine (Qiagen, Germany). The HTLV-I copy number was reported as an actual amount of cellular DNA by means of quantification of the albumin gene as the reference gene. HTLV-I and albumin DNA concentrations were calculated from two 5-point standard curves. The normalized value of the HTLV- I proviral load was calculated as the ratio of (HTLV-I DNA copies number/albumin DNA copies number/2)×10^4^ and expressed as the number of HTLV-I proviruses per 10^4^ PBMCs (15).

*Statistical analysis*

Data Analysis was performed by SPSS (version 18 ). Inferential statistical methods including Man Withney, T student and Wilcoxon tests were used to compare the differences between clinical groups and healthy control. Results were statistically significant if *P-* value was < 0.05 .

## Results

Proviral load of HTLV-I investigated in 47 patients, 11 healthy carriers with 38.65±14.9 (CI 95%: 8-48) years old, 6 males and 5 females, 23 HAM/TSP patients with 45.52±15.17 (CI 95%: 38.9 up to 52) years old 9 males and 14 females. Thirteen individual were candidate for ATL, which the disease confirmed in nine individuals according to symptoms and laboratory tests. The average age for ATL group was 52±8 (CI 95%: 45-58) years-old (3 males and 5 females).

The mean HTLV-I proviral load in ATL patients was 11697.2±5078 copies/10 ^4^(CI 95%:115-23707), in males 17195.67± 11925.36 (CI95%:2368-68076.2) copies/10 ^4^ and in females 8398.2±4797.99 (CI95%:1923-21719.57) copies/10^4^. The percentage of HTLV-I infected PBMCs in ATL group was 116.6±143 (CI 95%: 2.3-236.65), in male 171.31± 118 (CI 95%:137-680) and in female 83.8±47.82 (CI95%: 19.23-21.719).

In HAM/TSP patients mean HTLV-I proviral load was 409 ± 71.3 (CI: 95% 261-556) copies/10 ^4^, males proviral load average was 363 ± 113.9 (CI95%:3.66-632.3) copies/10 ^4^ and 438.36± 99.9 copies/10 ^4^ in females. In HAM/TSP patients the percentage of HTLV-I infected PBMCs was 3.6±1.14 % and 4.4±1% in male and female, respectively. 11Healthy Carriers HTLV-I proviral load average was 373.6 ± 143.3 (CI 95%:54.2-693) copies/ 10 ^4^ which 3.7± 1.4(CI 95%:0.5-6.9) % of PBMCs were infected. In healthy Carriers HTLV-I proviral load was 427.67 ± 230.825 copies/10 ^4^, in males 427.67 ± 230.825 and in females was 308.8± 176.51. The percentages of infected PBMCs were 4.25± 2.298 % and 3.08±1.76% in male and females, respectively.

**Table 1:** classified data of HTLV-I proviral load according to sex and groups of the patients

HAM/TSP			
sex	N	copies/10 ^4^	Infected cells%
Male	9	363±113.9 (93.66-632.3)	3.6±1.14 (0.9-6.29)
Female	14	438.36±99.9 (222.42-654)	4.4±1 (1.69-6.236)
Healthy Carriers			
Male	6	427.67±230.825 (160-1021)	4.27±2.298 (1.6-10.2)
Female	5	308.8±176.516 (181.29-798.86)	3.08±1.76 (1.82-7.99)
ATL			
Male	3	17195.67±11825.36 (2368-68076.3)	171.95±118 (236-680)
Female	5	8398.2±4797.99 (1923-21719.57)	83.8±47.82 19.23±21.719

Classified HTLV-I proviral load according to sex and groups of the patients is shown in Table 1The highest HTLV-I proviral load was measured in ATL group that had a significant correlation with WBC count (R=0.495, *P=*0.001). The proviral load variations between study groups was strongly significant (ATL *vs* carrier *P=*0.0001; ATL *vs* HAM/TSP *P=* 0.0001 and HAM/TSP *vs* carriers p< 0.05).

 Characteristics and proviral load of HAM/TSP, ATL patients and asymptomatic carrier’s subjects are summaDiscussion.

**Table 2:** Characteristics and proviral load of HAM/TSP, ATL patients and asymptomatic carrier’s subjects are summarized

Variables	ATL	HAM/TSP	Healthy carriers
Age (years)	52 ±8	45.52±15.17	38.65±14.9
WBC /ml	21550±15400	5584.3±903.2	5245.4±1034.7
Proviral load copies / 104	11697.2±5078	409±71.3	373.6±143.3
Infected cell (%)	116.6±50.74	4.09±0.73	3.7±1.4

In this study, HTLV-I proviral load investigated among three groups of HTLV-I infected, HAM/TSP, ATL and healthy carriers in northeast of Iran –Khorasan state. Viral factors, together with host genetics, are associated with an increased risk of developing HAM/TSP, ATL and clinical progression of these diseases that proved in previous studies (16). The most important factors are HTLV-I proviral load, HTLV-I subgroups, HLA background, frequency of HTLV-I-specific CD4^+^ T cells, age, and gender, routes of transmission (i.e., breastfeeding or transfusion), and high antibody titers against HTLV-I and TAX (16-18). HTLV-I proviral load considered as a main and smart factor in prognosis and development of HTLV-I associated diseases (19).

In the present study, there are significant variations between ATL, HAM/TSP and healthy carriers individuals for HTLV-I proviral load. Independent risk factors for the development of ATL, after adjusting for proviral load are increased age, family history of ATL, and first opportunity to learn HTLV-I infection during the treatment (20). ATL and Ham/TSP are the most important conditions of HTLV-I infection in individuals (1) however, a small number of infected HTLV-I developed these diseases and a large part of them remained healthy carriers. In this study, the lowest HTLV-I proviral load was observed in asymptomatic carriers which significantly differ from ATL and HAM/TSP groups. Furthermore, the highest HTLV-I proviral load was observed in ATL patients that had a significant difference with HAM/TSP individuals. Therefore, HTLV-I proviral load can be used as a differential prognostic factor for ATL. In addition, there was a significant correlation between proviral load and PBMCs count. Therefore, proviral load in association with lymphocyte count is a monitoring marker for progression and treatment of ATL. Suggested persistence of highest proviral load in HTLV-I associated diseases should happen in ascending of HTLV-I proviral load which Taylor *et al* reported the Lack of diversity in healthy carriers proviral loads (21).

The significant correlation with WBC count and HTLV-I proviral load in ATL patients in the present study could be proven by previous studies that showed decreasing count of T cell lymphocyte and HTLV-I proviral load in ATL patients after treatment (22-24).

 Previous studies reported no significant differences in the HTLV-I proviral load by sex and age in asymptomatic HTLV-I carriers (21, 25); result of our research demonstrated such outputs (Table 1). Some studies have shown higher load of provirus in infected HTLV-I with family history of leukemia and lymphoma (17). Other results claimed that the authors found a significant association between clinical status and HTLV-I proviral load among women (26). These results in asymptomatic carriers are in consistent with previous studies indicating lower HTLV-I proviral load than ATL and HAM/TSP patients (17-21).

In one study, authors found HTLV-I viral load difference in male and female, which might be the result of mode of transmission or acquisition of the virus and the methodology of study (27). In the present research, the mean HTLV-I proviral load between male and female was not significant in HAM/TSP and healthy carriers, whereas in ATL individuals, the mean HTLV-I proviral load in males was two times more than females (Table 1), although to prove this fact, complementary research with more population is recommended.

Entirely, HTLV-I proviral load is a valuable prognostic factor for development of HTLV-I associated diseases and can be used as a monitoring marker for the progression and efficiency of therapeutic regime.

## Conclusion

Results of the present study demonstrated that HTLV-I proviral load is a valuable marker for monitoring of ATL and HAM/TSP patients particularly during therapy. Moreover, as HTLV-I proviral load is highest in ATL patients and lowest in healthy carriers, this factor may influence on development and progression of HTLV-I associated diseases and can be used as a prognostic test in HTLV-I infected subjects.
